# Data on the gene expression of cardiomyocyte exposed to hypothermia

**DOI:** 10.1016/j.dib.2016.04.061

**Published:** 2016-05-11

**Authors:** Jian Zhang, Xiaodong Xue, Yinli Xu, Yuji Zhang, Zhi Li, Huishan Wang

**Affiliations:** Department of Cardiovascular Surgery, General Hospital of Shenyang Military Area Command, No. 83, Wenhua Road, Shenhe District, Shenyang City, Liaoning 110016, China

**Keywords:** Hypothermia, Cardiomyocyte, Transcriptome

## Abstract

Hypothermia is widely used in neurosurgery and cardiac surgeries. However, little is known about the underlying molecular mechanisms. We previously reported that the transcriptome responses of cardiomyocyte exposed to hypothermia, “The transcriptome responses of cardiomyocyte exposed to hypothermia” [4]. Herein, we provide the hypothermia inhibited proliferation of cardiomyocyte cells in vitro and the details of transcription factors in regulation of differentially expressed genes.

**Specifications Table**

**Table:** 

Subject area	Biology
More specific subject area	Hypothermia and cardiology
Type of data	Tables and figures
How data was acquired	Polymerase Chain Reaction (Applied Biosystems PCR System 7900); Affymetrix GeneChip HTA 2.0 arrays (Affymetrix, Santa Clara, USA) were hybridized with biotin-labeled RNA probes.
Data format	Analyzed
Experimental factors	Adult ventricular cardiomyocyte cells (AC16) was treated with hypothermia
Experimental features	Cells were cultured at 37 °C or 28 °C with 5% CO_2_ for 6 h
Data source location	Shenyang city, Liaoning province, China
Data accessibility	Data are presented in this article

**Value of the data**

• The data provides the inhibition of hypothermia on the cardiomyocytes in vitro culture.
• This data provides the details of differentially expressed genes (DEGs) of cardiomyocytes exposed on hypothermia.
• The data may stimulate further research on the function of transcription factor (TF) stimulated in cardiomyocytes under hypothermia.

## 1 Data

**Fig. 1:**
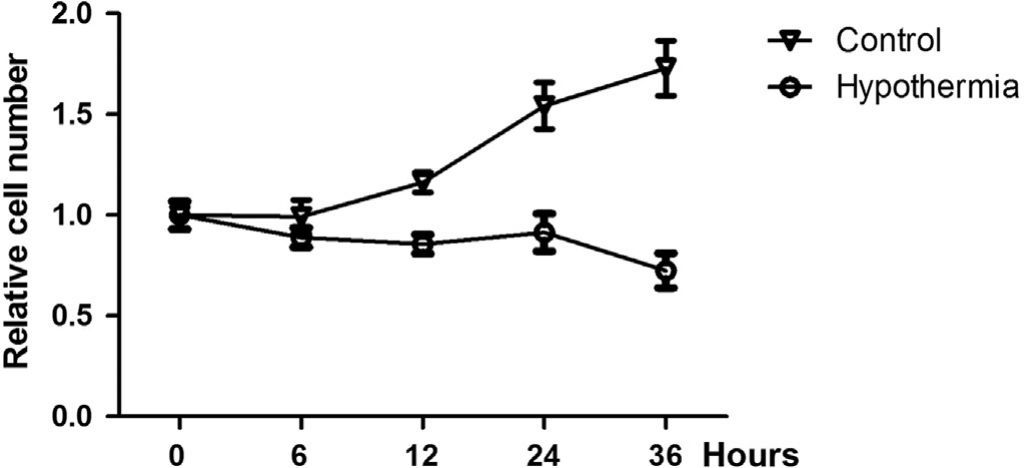
Hypothermia inhibited proliferation of cardiomyocyte cells. Cell proliferation was detected by CCK-8 assay at various time points according to the guidance of the manufacturer.

The viable cell number was determined by Cell Counting Kit-8 (CCK-8) assay (Fig. 1). As shown in Fig. 1, the relative cell number of hypothermia was decreased as the time of hypothermia culture.

The details of changed genes are listed in Supplementary Table S1.

Pathway enrichment analysis was performed considering the notion that different genes cooperate with each other to exercise their biological functions. The changed pathways are listed in Supplementary Table S2.

The details of TFs in regulation of DEGs are listed in Supplementary Table S3.

**Fig. 2:**
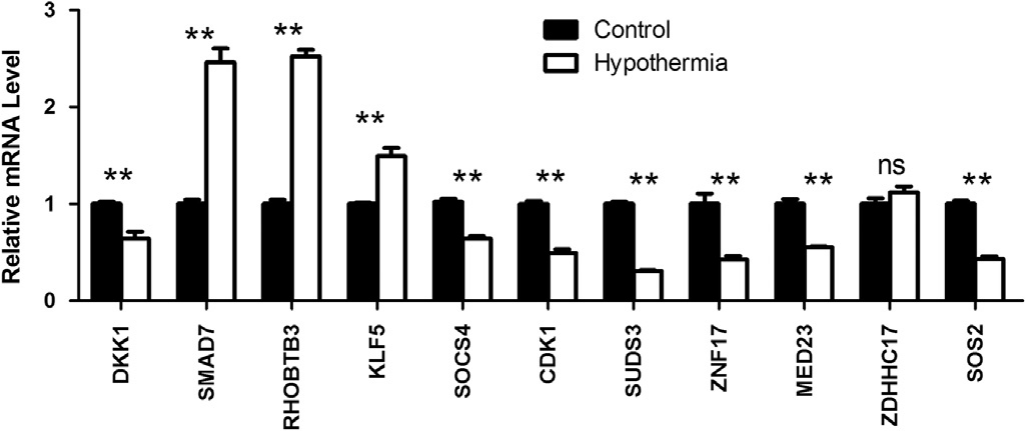
qRT-PCR validation of 11 differentially expressed genes during hypothermia stress. Dickkopf 1 homolog (DKK1), SMAD family member 7 (SMAD7), Rho-related BTB domain containing 3 (RHOBTB3), Kruppel-like factor 5 (KLF5), Suppressor of cytokine signaling 4 (SOCS4), Cyclin-dependent kinase 1 (CDK1), Suppressor of defective silencing 3 homolog(SUDS3), Zinc finger protein 17 (ZNF17), Mediator complex subunit 23 (MED23), Zinc finger, DHHC-type containing 17 (ZDHHC17), Son of sevenless homolog 2 (SOS2).

Primers for the 11 randomly selected differentially expressed genes are listed in Supplementary Table S4. The amount of 18s, a constitutive transcript (endogenous control) was normalized to check the fold change in the expression of the target genes (Fig. 2).

## 2 Experimental design, materials and methods

### 2.1 Experimental design and hypothermia treatment

AC16 human adult ventricular cardiomyocytes were cultured in incubator with normal temperature (37 °C) and 5% CO_2_. The hypothermia treated AC16 cells were cultured in another incubator with low temperature (28 °C) and 5% CO_2_. The cells incubated for six hours and the RNA was extracted using the TRIzol (Invitrogen) reagent. This experiment was repeated three times (*N*=3). Then, the RNA was isolated by Genminix Co. (Shanghai, China). Finally, the microarray hybridization was completed [4].

### 2.2 Cells culture

AC16 human adult ventricular cardiomyocytes were purchased from the American Type Cell Culture (ATCC) [2]. The cells were maintained in DMEM/F-12 supplemented with 10% fetal bovine serum, 100 units/ml penicillin, and 100 μg/ml streptomycin. Cells were cultured at 37 °C or 28 °C with 5% CO_2_.

### 2.3 Cell Counting Kit-8 (CCK8) assay

Cell viability was determined by the Cell Counting Kit-8 assay (DOJINDO, Japan), according to the manufacturer׳s instructions. The absorbance of each well was measured at 450 nm with a microtiter plate reader.

### 2.4 Microarray hybridization for the hypothermia regulated transcriptome

Microarray hybridization has been described previously in [4].

### 2.5 GO analysis

Gene Ontology (GO) analysis was applied to analyze the main function of the differentially expressed genes according to the gene ontology, which is the key functional classification of NCBI that can organize genes into hierarchical categories and uncover the gene regulatory network based on biological process and molecular function [1].

## 3 Pathway analysis

Pathway analysis was used to identify the significant pathway of the differential genes according to KEGG, Biocarta and Reactome. Fisher׳s exact test was used to select the significant pathway, and the threshold of significance was defined by *P*-value and FDR. The enrichment Re was calculated based on the previously published equation [3].

## 4 Statistical analysis

RVM *t*-test was applied to filter the differentially expressed genes for the control and hypothermia treated group because the RVM *t*-test can raise degrees of freedom effectively in the cases of small samples. After the significant analysis and FDR analysis, we selected the differentially expressed genes according to the *p*-value threshold. *P* value <0.05 was considered as significant difference [4].

## Footnotes

Appendix A Supplementary data associated with this article can be found in the online version at doi:10.1016/j.dib.2016.04.061.
