# Supplementary material for: Data on the gene expression of cardiomyocyte exposed to hypothermia
Source: Data Brief. 2016 May 11;8:45–8. doi: 10.1016/j.dib.2016.04.061 (PMC4885011; doi:10.1016/j.dib.2016.04.061)
Supplement: Supplementary file 1 — Supplementary material [file mmc1.doc]

**Title: Data on the gene expression of cardiomyocyte exposed to hypothermia**

**Competing Interests:** The authors have declared that no competing interests exist.
