# Supplementary material for: Data on the gene expression of cardiomyocyte exposed to hypothermia
Source: Data Brief. 2016 May 11;8:45–8. doi: 10.1016/j.dib.2016.04.061 (PMC4885011; doi:10.1016/j.dib.2016.04.061)
Supplement: Supplementary file 2 — Supplementary material [file mmc2.zip › supplementary data/Supplementary table 2.doc]

| **path_name** | **Diffgene count** | **Path gene count** | **enrichment** | **pvalue** | **FDR** |
| --- | --- | --- | --- | --- | --- |
| Pathways in cancer | 16 | 327 | 7.949983011 | 1.11037E-09 | 1.41017E-07 |
| Circadian rhythm | 6 | 31 | 31.44731183 | 1.33076E-07 | 8.4503E-06 |
| Osteoclast differentiation | 9 | 135 | 10.83185185 | 7.05454E-07 | 2.98642E-05 |
| Cytokine-cytokine receptor interaction | 11 | 267 | 6.693841032 | 3.90919E-06 | 0.000124117 |
| Transcriptional misregulation in cancer | 9 | 180 | 8.123888889 | 7.91799E-06 | 0.000201117 |
| Hippo signaling pathway | 8 | 156 | 8.332193732 | 2.45371E-05 | 0.000519369 |
| Proteoglycans in cancer | 9 | 227 | 6.44185022 | 5.18939E-05 | 0.000941503 |
| Rheumatoid arthritis | 6 | 94 | 10.37092199 | 0.000106416 | 0.001689353 |
| MAPK signaling pathway | 9 | 260 | 5.624230769 | 0.000150783 | 0.002127714 |
| HTLV-I infection | 9 | 268 | 5.456343284 | 0.000190623 | 0.002420908 |
| Jak-STAT signaling pathway | 7 | 158 | 7.19838256 | 0.000239379 | 0.002763744 |
| Bladder cancer | 4 | 38 | 17.10292398 | 0.000351406 | 0.003719045 |
| TGF-beta signaling pathway | 5 | 81 | 10.02949246 | 0.00059544 | 0.005816994 |
| Herpes simplex infection | 7 | 188 | 6.049704492 | 0.000706824 | 0.006411899 |
| Salmonella infection | 5 | 88 | 9.231691919 | 0.000879155 | 0.007006158 |
| Type II diabetes mellitus | 4 | 48 | 13.53981481 | 0.000882666 | 0.007006158 |
| Estrogen signaling pathway | 5 | 100 | 8.123888889 | 0.001591387 | 0.011888598 |
| Epithelial cell signaling in Helicobacter pylori infection | 4 | 68 | 9.55751634 | 0.003355205 | 0.023672834 |
| Amphetamine addiction | 4 | 70 | 9.284444444 | 0.003741426 | 0.025008481 |
| SNARE interactions in vesicular transport | 3 | 36 | 13.53981481 | 0.005666266 | 0.035980787 |
| Hepatitis B | 5 | 148 | 5.489114114 | 0.009204619 | 0.055666028 |
| Legionellosis | 3 | 55 | 8.862424242 | 0.019107017 | 0.110299596 |
| NOD-like receptor signaling pathway | 3 | 57 | 8.551461988 | 0.021119025 | 0.111754841 |
| Acute myeloid leukemia | 3 | 57 | 8.551461988 | 0.021119025 | 0.111754841 |
| Colorectal cancer | 3 | 62 | 7.861827957 | 0.026690153 | 0.135585975 |
| Synaptic vesicle cycle | 3 | 64 | 7.616145833 | 0.029139814 | 0.142336781 |
| Pancreatic cancer | 3 | 66 | 7.385353535 | 0.031718347 | 0.149193707 |
| Maturity onset diabetes of the young | 2 | 25 | 12.99822222 | 0.041252347 | 0.187108859 |
| Insulin signaling pathway | 4 | 140 | 4.642222222 | 0.044805793 | 0.190303315 |
| Pertussis | 3 | 75 | 6.499111111 | 0.044953539 | 0.190303315 |
| Wnt signaling pathway | 4 | 143 | 4.544832945 | 0.048128412 | 0.197171238 |
|  |  |  |  |  |  |

**Table 2. KEGG pathway enrichment analysis of DEGs (Up-regulated)**

KEGG pathway enrichment analysis of DEGs (Down-regulated)

| **path_name** | **Diffgene count** | **Path gene count** | **enrichment** | **pvalue** | **FDR** |
| --- | --- | --- | --- | --- | --- |
| Fanconi anemia pathway | 17 | 54 | 9.971560369 | 1.74657E-12 | 3.87738E-10 |
| Ubiquitin mediated proteolysis | 23 | 138 | 5.279061372 | 2.9087E-10 | 3.22866E-08 |
| RNA degradation | 15 | 72 | 6.598826715 | 2.44647E-08 | 1.50266E-06 |
| Chronic myeloid leukemia | 15 | 73 | 6.508431828 | 2.98955E-08 | 1.50266E-06 |
| Epstein-Barr virus infection | 25 | 204 | 3.881662773 | 3.38438E-08 | 1.50266E-06 |
| Cell cycle | 19 | 124 | 4.853330616 | 5.3687E-08 | 1.98642E-06 |
| TGF-beta signaling pathway | 15 | 81 | 5.865623746 | 1.32199E-07 | 4.1926E-06 |
| Viral carcinogenesis | 24 | 207 | 3.67239052 | 1.96114E-07 | 5.44217E-06 |
| Small cell lung cancer | 15 | 86 | 5.52459911 | 3.05123E-07 | 7.52637E-06 |
| Renal cell carcinoma | 13 | 66 | 6.238890712 | 5.05536E-07 | 1.12229E-05 |
| Neurotrophin signaling pathway | 17 | 120 | 4.487202166 | 9.88804E-07 | 1.92946E-05 |
| MAPK signaling pathway | 26 | 260 | 3.167436823 | 1.0898E-06 | 1.92946E-05 |
| T cell receptor signaling pathway | 16 | 108 | 4.692498997 | 1.18719E-06 | 1.92946E-05 |
| RNA transport | 20 | 165 | 3.839317361 | 1.21678E-06 | 1.92946E-05 |
| Ribosome biogenesis in eukaryotes | 14 | 84 | 5.279061372 | 1.47799E-06 | 2.18743E-05 |
| Insulin signaling pathway | 18 | 140 | 4.072418773 | 1.94861E-06 | 2.7037E-05 |
| Apoptosis | 14 | 88 | 5.039104037 | 2.66374E-06 | 3.47853E-05 |
| Measles | 17 | 134 | 4.018389999 | 4.85488E-06 | 5.98769E-05 |
| Nucleotide excision repair | 10 | 47 | 6.739227283 | 6.80503E-06 | 7.59774E-05 |
| Homologous recombination | 8 | 28 | 9.049819495 | 6.84481E-06 | 7.59774E-05 |
| PI3K-Akt signaling pathway | 29 | 347 | 2.647137403 | 9.54403E-06 | 9.79973E-05 |
| Herpes simplex infection | 20 | 188 | 3.369613642 | 9.71144E-06 | 9.79973E-05 |
| ErbB signaling pathway | 13 | 88 | 4.679168034 | 1.56161E-05 | 0.000150729 |
| Hepatitis B | 17 | 148 | 3.638272027 | 1.9293E-05 | 0.000177395 |
| Hepatitis C | 16 | 133 | 3.810450314 | 1.99769E-05 | 0.000177395 |
| Chagas disease (American trypanosomiasis) | 14 | 105 | 4.223249097 | 2.30969E-05 | 0.000197212 |
| mRNA surveillance pathway | 13 | 92 | 4.475725946 | 2.58472E-05 | 0.000207523 |
| Pathways in cancer | 27 | 327 | 2.615314808 | 2.61741E-05 | 0.000207523 |
| Toxoplasmosis | 15 | 122 | 3.894389537 | 2.99099E-05 | 0.000228965 |
| Basal transcription factors | 9 | 45 | 6.334873646 | 3.86133E-05 | 0.000285739 |
| Acute myeloid leukemia | 10 | 57 | 5.556906707 | 4.26286E-05 | 0.000305275 |
| Jak-STAT signaling pathway | 17 | 158 | 3.408001645 | 4.64704E-05 | 0.000322389 |
| Influenza A | 18 | 179 | 3.185132001 | 6.6203E-05 | 0.000445365 |
| mTOR signaling pathway | 10 | 60 | 5.279061372 | 6.8314E-05 | 0.00044605 |
| Metabolic pathways | 65 | 1189 | 1.731567649 | 8.91278E-05 | 0.000565325 |
| Prostate cancer | 12 | 89 | 4.270701335 | 9.43794E-05 | 0.000576418 |
| Protein processing in endoplasmic reticulum | 17 | 167 | 3.224336886 | 9.60697E-05 | 0.000576418 |
| Proteoglycans in cancer | 20 | 227 | 2.790693236 | 0.000159276 | 0.000917004 |
| Pancreatic cancer | 10 | 66 | 4.799146702 | 0.000161095 | 0.000917004 |
| Epithelial cell signaling in Helicobacter pylori infection | 10 | 68 | 4.657995328 | 0.000209708 | 0.001163881 |
| Spliceosome | 14 | 132 | 3.359402691 | 0.000313567 | 0.001697852 |
| Progesterone-mediated oocyte maturation | 11 | 86 | 4.051372681 | 0.000334474 | 0.001767934 |
| Osteoclast differentiation | 14 | 135 | 3.284749298 | 0.00040013 | 0.002065787 |
| Inositol phosphate metabolism | 9 | 61 | 4.673267444 | 0.000480787 | 0.002425788 |
| Colorectal cancer | 9 | 62 | 4.597892163 | 0.000546804 | 0.002646253 |
| B cell receptor signaling pathway | 10 | 76 | 4.16768003 | 0.000548323 | 0.002646253 |
| Toll-like receptor signaling pathway | 12 | 108 | 3.519374248 | 0.000640968 | 0.003027549 |
| Regulation of actin cytoskeleton | 18 | 215 | 2.651807573 | 0.000721963 | 0.003339078 |
| Transcriptional misregulation in cancer | 16 | 180 | 2.815499398 | 0.000820331 | 0.0037166 |
| Tuberculosis | 16 | 184 | 2.75429289 | 0.001053431 | 0.004677234 |
| Non-small cell lung cancer | 8 | 54 | 4.692498997 | 0.00110759 | 0.004821272 |
| Endocytosis | 17 | 204 | 2.639530686 | 0.001138128 | 0.004858932 |
| Legionellosis | 8 | 55 | 4.607180834 | 0.001260576 | 0.005244461 |
| Focal adhesion | 17 | 206 | 2.613904174 | 0.00127568 | 0.005244461 |
| Circadian rhythm | 6 | 31 | 6.130522883 | 0.00146486 | 0.005912707 |
| NOD-like receptor signaling pathway | 8 | 57 | 4.445525366 | 0.001618309 | 0.006415438 |
| Olfactory transduction | 2 | 408 | 0.155266511 | 0.001952188 | 0.007603259 |
| HTLV-I infection | 20 | 268 | 2.363758823 | 0.002220197 | 0.008497994 |
| RNA polymerase | 6 | 34 | 5.589594394 | 0.002468378 | 0.009287796 |
| Mismatch repair | 5 | 23 | 6.885732224 | 0.002607056 | 0.009646106 |
| Axon guidance | 12 | 131 | 2.901468846 | 0.003769117 | 0.013717115 |
| Glioma | 8 | 65 | 3.898383782 | 0.003959971 | 0.014179251 |
| Phosphatidylinositol signaling system | 9 | 81 | 3.519374248 | 0.004125222 | 0.014309364 |
| Peroxisome | 9 | 81 | 3.519374248 | 0.004125222 | 0.014309364 |
| Endometrial cancer | 7 | 52 | 4.263857262 | 0.004764057 | 0.016271088 |
| Estrogen signaling pathway | 10 | 100 | 3.167436823 | 0.005041166 | 0.01695665 |
| Adipocytokine signaling pathway | 8 | 71 | 3.568942899 | 0.007071745 | 0.023431751 |
| Alcoholism | 14 | 180 | 2.463561974 | 0.007299015 | 0.023829138 |
| Wnt signaling pathway | 12 | 143 | 2.657988942 | 0.008009936 | 0.025771099 |
| Adherens junction | 8 | 73 | 3.471163642 | 0.008455365 | 0.026815586 |
| NF-kappa B signaling pathway | 9 | 92 | 3.098579501 | 0.010105913 | 0.03159877 |
| Neuroactive ligand-receptor interaction | 1 | 275 | 0.115179521 | 0.010946659 | 0.0337522 |
| Oocyte meiosis | 10 | 112 | 2.828068592 | 0.011759662 | 0.035565695 |
| Bacterial invasion of epithelial cells | 8 | 77 | 3.290843453 | 0.011855232 | 0.035565695 |
| Regulation of autophagy | 5 | 34 | 4.657995328 | 0.016183253 | 0.047815557 |
| Hippo signaling pathway | 12 | 156 | 2.436489864 | 0.01636929 | 0.047815557 |
| p53 signaling pathway | 7 | 68 | 3.26059673 | 0.022359614 | 0.06446538 |
| Fc epsilon RI signaling pathway | 7 | 70 | 3.167436823 | 0.026195466 | 0.074556327 |
| Pyrimidine metabolism | 9 | 107 | 2.664199197 | 0.027505866 | 0.076582212 |
| Cytokine-cytokine receptor interaction | 17 | 267 | 2.016720075 | 0.028009764 | 0.076582212 |
| RIG-I-like receptor signaling pathway | 7 | 71 | 3.122825037 | 0.028287123 | 0.076582212 |
| Melanoma | 7 | 71 | 3.122825037 | 0.028287123 | 0.076582212 |
| Glycosylphosphatidylinositol(GPI)-anchor biosynthesis | 4 | 25 | 5.067898917 | 0.029473469 | 0.078832652 |
| Ether lipid metabolism | 5 | 42 | 3.770758123 | 0.040231555 | 0.106326253 |
| Chemokine signaling pathway | 13 | 192 | 2.144618682 | 0.041542535 | 0.108499326 |
| Shigellosis | 6 | 61 | 3.115511629 | 0.04968124 | 0.128246921 |
