# Supplementary material for: Data on the gene expression of cardiomyocyte exposed to hypothermia
Source: Data Brief. 2016 May 11;8:45–8. doi: 10.1016/j.dib.2016.04.061 (PMC4885011; doi:10.1016/j.dib.2016.04.061)
Supplement: Supplementary file 2 — Supplementary material [file mmc2.zip › supplementary data/Supplementary table 3.doc]

Table.3 The details of TFs in regulation of DEGs

| **TF** | **Genesymbol** |
| --- | --- |
| ZNF333 | RANBP2 |
| ZNF333 | ATF2 |
| ZNF333 | DUSP5 |
| ZNF333 | DHX36 |
| ZNF333 | TAOK3 |
| ZNF333 | PRKCI |
| ZNF333 | PIAS1 |
| ZNF333 | TEC |
| ZNF333 | BTRC |
| ZNF333 | GEMIN5 |
| ZNF333 | CDKN2B |
| ZNF333 | NF1 |
| ZNF333 | ERCC8 |
| ZNF333 | ROCK2 |
| ZNF333 | CDC27 |
| ZNF333 | GADD45A |
| ZNF333 | DBF4 |
| ZNF333 | LSM6 |
| ZNF333 | EXOSC2 |
| ZNF333 | CNOT1 |
| ZNF333 | SOS1 |
| ZNF333 | RB1 |
| ZNF333 | SOS2 |
| ZNF333 | ANAPC4 |
| ZNF333 | FOXO1 |
| ZNF333 | IRAK4 |
| ZNF333 | BIRC6 |
| ZNF333 | ID4 |
| ZNF333 | BIRC3 |
| ZNF333 | UBA6 |
| ZNF333 | NCBP1 |
| ZNF333 | HEATR1 |
| ZNF333 | PPP1R3C |
| ZNF333 | CXCL2 |
| ZNF333 | KLF3 |
| ZNF333 | EIF5B |
| ZNF333 | NUP153 |
| ZNF333 | AKT3 |
| ZNF333 | PAK6 |
| ZNF333 | PIK3CB |
| ZNF333 | BMP4 |
| ZNF333 | NUP43 |
| ZNF333 | ORC5 |
| ZNF333 | TRIP12 |
| ZNF333 | FANCL |
| ZNF333 | DUSP1 |
| ZNF333 | SKP2 |
| ZNF333 | THOC1 |
| ZNF333 | RUNX1 |
| ZNF333 | CHUK |
| ZNF333 | RBL2 |
| ZNF333 | ID3 |
| ZNF333 | CDK1 |
| ZNF333 | TSC1 |
| ZNF333 | CTGF |
| ZNF333 | CDK7 |
| ZNF333 | CBLB |
| ZNF333 | CUL4B |
| ZNF333 | NAT10 |
| ZNF333 | NVL |
| ZNF333 | ID1 |
| ZNF333 | NUP160 |
| ZNF333 | RPS6KB1 |
| ZNF333 | UBE3C |
| ZNF333 | NUP107 |
| ZNF333 | FRS2 |
| ZNF333 | PLAUR |
| ZNF333 | THOC2 |
| ZNF333 | XRN1 |
| ZNF333 | DCP1B |
| ZNF333 | NFKB1 |
| ZNF333 | E2F4 |
| ZNF333 | POP1 |
| ZNF333 | CUL1 |
| ZNF333 | BHLHE41 |
| ZNF333 | NFKBIE |
| ZNF333 | RIOK1 |
| ZNF333 | TGS1 |
| ZNF333 | FZD8 |
| ZNF333 | PIK3CA |
| ZNF333 | CRY2 |
| ZNF333 | ETV4 |
| ZNF333 | SMAD2 |
| ZNF333 | DDX20 |
| ZNF333 | FADD |
| ZNF333 | ITCH |
| ZNF333 | MAP3K7 |
| ZNF333 | NUPL1 |
| ZNF333 | PRKAA2 |
| ZNF333 | BUB1 |
| ZNF333 | CNOT6 |
| YY1 | TAOK3 |
| YY1 | PIM1 |
| YY1 | CDKN2B |
| YY1 | CDC27 |
| YY1 | LSM6 |
| YY1 | CNOT1 |
| YY1 | BIRC3 |
| YY1 | ATR |
| YY1 | NUP153 |
| YY1 | UPF2 |
| YY1 | NUP43 |
| YY1 | SPRY2 |
| YY1 | CHUK |
| YY1 | ID3 |
| YY1 | CDK7 |
| YY1 | UBE3A |
| YY1 | MAP3K1 |
| YY1 | NVL |
| YY1 | ID1 |
| YY1 | SPRY4 |
| YY1 | SPATA5 |
| YY1 | UBE3C |
| YY1 | DCP2 |
| YY1 | NUP50 |
| YY1 | PLAUR |
| YY1 | IRS2 |
| YY1 | E2F4 |
| YY1 | TGS1 |
| YY1 | PAK2 |
| YY1 | NUP133 |
| YY1 | MAP3K7 |
| YY1 | CNOT6 |
| TTF-1 | BTRC |
| TTF-1 | CLCF1 |
| TTF-1 | NGF |
| TTF-1 | LATS2 |
| TTF-1 | LSM6 |
| TTF-1 | AKT3 |
| TTF-1 | SPRY2 |
| TTF-1 | ORC5 |
| TTF-1 | FANCL |
| TTF-1 | CUL4B |
| TTF-1 | NVL |
| TTF-1 | SPRY4 |
| TTF-1 | ID2 |
| TTF-1 | NUP107 |
| TTF-1 | THOC2 |
| TTF-1 | DCP1B |
| TTF-1 | BHLHE41 |
| TTF-1 | BHLHE40 |
| TTF-1 | CDKN1B |
| TTF-1 | SMAD2 |
| TTF-1 | NUPL1 |
| STAT1 | ANAPC4 |
| STAT1 | JUN |
| SRY | TOB2 |
| SRY | DHX36 |
| SRY | PRKCI |
| SRY | PIAS1 |
| SRY | BTRC |
| SRY | GEMIN5 |
| SRY | PIM1 |
| SRY | CDKN2B |
| SRY | CLCF1 |
| SRY | NF1 |
| SRY | LATS2 |
| SRY | GADD45A |
| SRY | LSM6 |
| SRY | EXOSC2 |
| SRY | CNOT1 |
| SRY | RB1 |
| SRY | SMURF2 |
| SRY | SOS2 |
| SRY | FOXO1 |
| SRY | ACVR2A |
| SRY | IRAK4 |
| SRY | BIRC6 |
| SRY | ID4 |
| SRY | MMP2 |
| SRY | BIRC3 |
| SRY | HSPA1A |
| SRY | APAF1 |
| SRY | NCBP1 |
| SRY | HEATR1 |
| SRY | CXCL2 |
| SRY | EIF5B |
| SRY | SMAD5 |
| SRY | AKT3 |
| SRY | PAK6 |
| SRY | PIK3CB |
| SRY | BMP4 |
| SRY | PER2 |
| SRY | NUP43 |
| SRY | SPRY2 |
| SRY | ORC5 |
| SRY | PHKB |
| SRY | DUSP1 |
| SRY | RUNX1 |
| SRY | CASP3 |
| SRY | CHUK |
| SRY | RBL2 |
| SRY | CDK1 |
| SRY | TSC1 |
| SRY | TGFB2 |
| SRY | JUN |
| SRY | NR1D1 |
| SRY | RPS6KA5 |
| SRY | RASA1 |
| SRY | CDK7 |
| SRY | CBLB |
| SRY | CUL4B |
| SRY | MAP3K1 |
| SRY | ORC2 |
| SRY | SMAD6 |
| SRY | NVL |
| SRY | SPATA5 |
| SRY | NUP160 |
| SRY | ID2 |
| SRY | DCP2 |
| SRY | BMPR1A |
| SRY | NUP107 |
| SRY | FRS2 |
| SRY | PLAUR |
| SRY | THOC2 |
| SRY | HSPG2 |
| SRY | XRN1 |
| SRY | DCP1B |
| SRY | IRS2 |
| SRY | VEGFA |
| SRY | E2F4 |
| SRY | POP1 |
| SRY | CUL1 |
| SRY | BHLHE41 |
| SRY | NFKBIE |
| SRY | RIOK1 |
| SRY | PAK2 |
| SRY | FZD8 |
| SRY | CDKN1B |
| SRY | PIK3CA |
| SRY | NUP133 |
| SRY | CRY2 |
| SRY | ETV4 |
| SRY | DDX20 |
| SRY | FADD |
| SRY | PRKAA2 |
| SRY | TAOK1 |
| SRY | BUB1 |
| SRY | CNOT6 |
| Sp1 | TOB2 |
| Sp1 | DUSP5 |
| Sp1 | FZD5 |
| Sp1 | PIAS1 |
| Sp1 | PAPD7 |
| Sp1 | TEC |
| Sp1 | MPHOSPH6 |
| Sp1 | PIM1 |
| Sp1 | NLK |
| Sp1 | ROCK2 |
| Sp1 | DBF4 |
| Sp1 | JUND |
| Sp1 | SMURF2 |
| Sp1 | SOS2 |
| Sp1 | ANAPC4 |
| Sp1 | IRAK4 |
| Sp1 | ATR |
| Sp1 | KLF3 |
| Sp1 | UPF2 |
| Sp1 | CNOT6L |
| Sp1 | TRIP12 |
| Sp1 | CASP3 |
| Sp1 | SOCS3 |
| Sp1 | CRK |
| Sp1 | TSC1 |
| Sp1 | CBLB |
| Sp1 | UBE3A |
| Sp1 | INHBB |
| Sp1 | NUP160 |
| Sp1 | BMPR1A |
| Sp1 | RARA |
| Sp1 | FRS2 |
| Sp1 | XRN1 |
| Sp1 | IRS2 |
| Sp1 | BHLHE40 |
| Sp1 | NFKBIE |
| Sp1 | PAK2 |
| Sp1 | CDKN1B |
| Sp1 | NFAT5 |
| Sp1 | PRKAA2 |
| Sp1 | CNOT6 |
| Smad4 | DUSP5 |
| Smad4 | PAPD7 |
| Smad4 | CXCL2 |
| Smad4 | NUP153 |
| Smad4 | PIK3CB |
| Smad4 | BMP4 |
| Smad4 | RBL2 |
| Smad4 | TGFB2 |
| Smad4 | RPS6KA5 |
| Smad4 | CUL4B |
| Smad4 | MAP3K1 |
| Smad4 | PLAUR |
| Smad4 | HSPG2 |
| Smad4 | DCP1B |
| Smad4 | BHLHE40 |
| Smad4 | PIK3R3 |
| Smad4 | ETV4 |
| Smad4 | NUPL1 |
| Smad4 | ATM |
| Smad4 | RFWD2 |
| RUSH-1alpha | NUP153 |
| RUSH-1alpha | AKT3 |
| RUSH-1alpha | ITCH |
| RUSH-1alpha | PRKAA2 |
| RNF96 | PIAS1 |
| RNF96 | PAPD7 |
| RNF96 | SOCS3 |
| RNF96 | CRK |
| RNF96 | SMAD6 |
| RNF96 | INHBB |
| RNF96 | FRS2 |
| RNF96 | IRS2 |
| RNF96 | TGS1 |
| RNF96 | SMAD2 |
| RNF96 | BRAF |
| RNF96 | TAOK1 |
| RFX | PLAUR |
| RelA-p65 | CXCL2 |
| Pit-1 | NF1 |
| NF-AT1 | ATF2 |
| NF-AT1 | TAOK3 |
| NF-AT1 | PRKCI |
| NF-AT1 | PIAS1 |
| NF-AT1 | TEC |
| NF-AT1 | BTRC |
| NF-AT1 | PIM1 |
| NF-AT1 | CDKN2B |
| NF-AT1 | CNOT4 |
| NF-AT1 | CLCF1 |
| NF-AT1 | ERCC8 |
| NF-AT1 | NLK |
| NF-AT1 | ROCK2 |
| NF-AT1 | CDC27 |
| NF-AT1 | GADD45A |
| NF-AT1 | DBF4 |
| NF-AT1 | LSM6 |
| NF-AT1 | SOS1 |
| NF-AT1 | RB1 |
| NF-AT1 | SMURF2 |
| NF-AT1 | SOS2 |
| NF-AT1 | ANAPC4 |
| NF-AT1 | FOXO1 |
| NF-AT1 | ID4 |
| NF-AT1 | MMP2 |
| NF-AT1 | BIRC3 |
| NF-AT1 | ATR |
| NF-AT1 | UBA6 |
| NF-AT1 | HSPA1A |
| NF-AT1 | APAF1 |
| NF-AT1 | MAFA |
| NF-AT1 | NCBP1 |
| NF-AT1 | HEATR1 |
| NF-AT1 | EIF5B |
| NF-AT1 | NUP153 |
| NF-AT1 | FST |
| NF-AT1 | AKT3 |
| NF-AT1 | PAK6 |
| NF-AT1 | PIK3CB |
| NF-AT1 | BMP4 |
| NF-AT1 | PER2 |
| NF-AT1 | UPF2 |
| NF-AT1 | SPRY2 |
| NF-AT1 | ORC5 |
| NF-AT1 | PHKB |
| NF-AT1 | TRIP12 |
| NF-AT1 | FANCL |
| NF-AT1 | DUSP1 |
| NF-AT1 | RPS6KA3 |
| NF-AT1 | SKP2 |
| NF-AT1 | THOC1 |
| NF-AT1 | RUNX1 |
| NF-AT1 | CASP3 |
| NF-AT1 | CHUK |
| NF-AT1 | SOCS3 |
| NF-AT1 | ID3 |
| NF-AT1 | DUSP2 |
| NF-AT1 | CDK1 |
| NF-AT1 | TGFB2 |
| NF-AT1 | JUN |
| NF-AT1 | NR1D1 |
| NF-AT1 | CTGF |
| NF-AT1 | RPS6KA5 |
| NF-AT1 | RASA1 |
| NF-AT1 | CDK7 |
| NF-AT1 | UBE3A |
| NF-AT1 | CUL4B |
| NF-AT1 | MAP3K1 |
| NF-AT1 | ACACA |
| NF-AT1 | ORC2 |
| NF-AT1 | SMAD6 |
| NF-AT1 | NVL |
| NF-AT1 | ID1 |
| NF-AT1 | SPRY4 |
| NF-AT1 | SPATA5 |
| NF-AT1 | NUP160 |
| NF-AT1 | RPS6KB1 |
| NF-AT1 | ID2 |
| NF-AT1 | MAPK14 |
| NF-AT1 | DCP2 |
| NF-AT1 | BMPR1A |
| NF-AT1 | NUP107 |
| NF-AT1 | FRS2 |
| NF-AT1 | PLAUR |
| NF-AT1 | THOC2 |
| NF-AT1 | HSPG2 |
| NF-AT1 | XRN1 |
| NF-AT1 | DCP1B |
| NF-AT1 | IRS2 |
| NF-AT1 | E2F4 |
| NF-AT1 | BHLHE41 |
| NF-AT1 | RIOK1 |
| NF-AT1 | FZD8 |
| NF-AT1 | PIK3CA |
| NF-AT1 | SMAD7 |
| NF-AT1 | PIK3R3 |
| NF-AT1 | NUP133 |
| NF-AT1 | CRY2 |
| NF-AT1 | EXOSC3 |
| NF-AT1 | NFAT5 |
| NF-AT1 | DDX20 |
| NF-AT1 | FADD |
| NF-AT1 | ITCH |
| NF-AT1 | MAP3K7 |
| NF-AT1 | NUPL1 |
| NF-AT1 | PRKAA2 |
| NF-AT1 | ATM |
| NF-AT1 | BRAF |
| NF-AT1 | TAOK1 |
| NF-AT1 | BUB1 |
| NF-AT1 | CNOT6 |
| NF-1A | RANBP2 |
| NF-1A | CDKN2B |
| NF-1A | GADD45A |
| NF-1A | CNOT1 |
| NF-1A | APAF1 |
| NF-1A | CXCL2 |
| NF-1A | AKT3 |
| NF-1A | PAK6 |
| NF-1A | PER2 |
| NF-1A | CDK7 |
| NF-1A | NAT10 |
| NF-1A | SPRY4 |
| NF-1A | SPATA5 |
| NF-1A | ID2 |
| NF-1A | THOC2 |
| NF-1A | DCP1B |
| NF-1A | CNOT6 |
| MZF-1 | PRKCI |
| MZF-1 | PIAS1 |
| MZF-1 | TEC |
| MZF-1 | PIM1 |
| MZF-1 | CDKN2B |
| MZF-1 | CLCF1 |
| MZF-1 | NF1 |
| MZF-1 | NLK |
| MZF-1 | NGF |
| MZF-1 | CDC27 |
| MZF-1 | LATS2 |
| MZF-1 | GADD45A |
| MZF-1 | EXOSC2 |
| MZF-1 | SOS1 |
| MZF-1 | JUND |
| MZF-1 | FOXO1 |
| MZF-1 | IRAK4 |
| MZF-1 | BIRC6 |
| MZF-1 | HSPA1A |
| MZF-1 | APAF1 |
| MZF-1 | HEATR1 |
| MZF-1 | FST |
| MZF-1 | SMAD5 |
| MZF-1 | PIK3CB |
| MZF-1 | PER2 |
| MZF-1 | SPRY2 |
| MZF-1 | PHKB |
| MZF-1 | FANCL |
| MZF-1 | RPS6KA3 |
| MZF-1 | RBL2 |
| MZF-1 | ID3 |
| MZF-1 | DUSP2 |
| MZF-1 | TGFB2 |
| MZF-1 | NR1D1 |
| MZF-1 | CTGF |
| MZF-1 | RPS6KA5 |
| MZF-1 | RASA1 |
| MZF-1 | CDK7 |
| MZF-1 | MAP3K1 |
| MZF-1 | INHBB |
| MZF-1 | NUP160 |
| MZF-1 | UBE3C |
| MZF-1 | BMPR1A |
| MZF-1 | RARA |
| MZF-1 | HSPG2 |
| MZF-1 | VEGFA |
| MZF-1 | E2F4 |
| MZF-1 | POP1 |
| MZF-1 | BHLHE40 |
| MZF-1 | NFKBIE |
| MZF-1 | CDKN1B |
| MZF-1 | SMAD7 |
| MZF-1 | PIK3R3 |
| MZF-1 | NUP133 |
| MZF-1 | SMAD2 |
| MZF-1 | NUPL1 |
| MZF-1 | ATM |
| MZF-1 | BUB1 |
| myogenin | PIAS1 |
| myogenin | GEMIN5 |
| myogenin | MPHOSPH6 |
| myogenin | CDKN2B |
| myogenin | CNOT4 |
| myogenin | NF1 |
| myogenin | GADD45A |
| myogenin | DBF4 |
| myogenin | EXOSC2 |
| myogenin | SOS2 |
| myogenin | ANAPC4 |
| myogenin | FOXO1 |
| myogenin | BIRC6 |
| myogenin | ID4 |
| myogenin | APAF1 |
| myogenin | MAFA |
| myogenin | NCBP1 |
| myogenin | PPP1R3C |
| myogenin | EIF5B |
| myogenin | FST |
| myogenin | SMAD5 |
| myogenin | AKT3 |
| myogenin | PAK6 |
| myogenin | PIK3CB |
| myogenin | TRIP12 |
| myogenin | DUSP1 |
| myogenin | RPS6KA3 |
| myogenin | SKP2 |
| myogenin | THOC1 |
| myogenin | CHUK |
| myogenin | RBL2 |
| myogenin | ID3 |
| myogenin | CRK |
| myogenin | CDK1 |
| myogenin | TSC1 |
| myogenin | TGFB2 |
| myogenin | JUN |
| myogenin | RASA1 |
| myogenin | UBE3A |
| myogenin | MAP3K1 |
| myogenin | NAT10 |
| myogenin | ORC2 |
| myogenin | ID1 |
| myogenin | SPRY4 |
| myogenin | SPATA5 |
| myogenin | RPS6KB1 |
| myogenin | ID2 |
| myogenin | MAPK14 |
| myogenin | BMPR1A |
| myogenin | RARA |
| myogenin | NUP107 |
| myogenin | NUP50 |
| myogenin | NFKB1 |
| myogenin | VEGFA |
| myogenin | E2F4 |
| myogenin | BHLHE40 |
| myogenin | NUP133 |
| myogenin | CRY2 |
| myogenin | ETV4 |
| myogenin | FADD |
| myogenin | PRKAA2 |
| myogenin | RFWD2 |
| myogenin | BUB1 |
| myogenin | PER1 |
| myogenin | CNOT6 |
| mef-2A | PIAS1 |
| MAZ | TOB2 |
| MAFA-TF | RANBP2 |
| MAFA-TF | ATF2 |
| MAFA-TF | DUSP5 |
| MAFA-TF | PIAS1 |
| MAFA-TF | PAPD7 |
| MAFA-TF | MPHOSPH6 |
| MAFA-TF | NF1 |
| MAFA-TF | NLK |
| MAFA-TF | EXOSC2 |
| MAFA-TF | SOS1 |
| MAFA-TF | RB1 |
| MAFA-TF | JUND |
| MAFA-TF | IRAK4 |
| MAFA-TF | ID4 |
| MAFA-TF | CXCL2 |
| MAFA-TF | PIK3CB |
| MAFA-TF | ORC5 |
| MAFA-TF | TRIP12 |
| MAFA-TF | SKP2 |
| MAFA-TF | RUNX1 |
| MAFA-TF | DUSP2 |
| MAFA-TF | TSC1 |
| MAFA-TF | TGFB2 |
| MAFA-TF | RASA1 |
| MAFA-TF | MAP3K1 |
| MAFA-TF | NAT10 |
| MAFA-TF | ID2 |
| MAFA-TF | FOS |
| MAFA-TF | PIK3R3 |
| MAFA-TF | EXOSC3 |
| MAFA-TF | ITCH |
| LEF-1 | DUSP5 |
| LEF-1 | PIM1 |
| LEF-1 | NF1 |
| LEF-1 | NLK |
| LEF-1 | NGF |
| LEF-1 | EXOSC2 |
| LEF-1 | CNOT1 |
| LEF-1 | BIRC3 |
| LEF-1 | PIK3CB |
| LEF-1 | NUP43 |
| LEF-1 | SPRY2 |
| LEF-1 | ORC5 |
| LEF-1 | DUSP2 |
| LEF-1 | NVL |
| LEF-1 | SPRY4 |
| LEF-1 | DCP2 |
| LEF-1 | THOC2 |
| LEF-1 | DCP1B |
| LEF-1 | NFKBIE |
| LEF-1 | TGS1 |
| LEF-1 | PIK3CA |
| LEF-1 | ATM |
| LEF-1 | PER1 |
| Kaiso | NUP133 |
| ipf1 | PIAS1 |
| ipf1 | TEC |
| ipf1 | CDC27 |
| ipf1 | LSM6 |
| ipf1 | MMP2 |
| ipf1 | ORC5 |
| ipf1 | CHUK |
| ipf1 | NAT10 |
| ipf1 | HSPG2 |
| ipf1 | POP1 |
| ING4 | RANBP2 |
| ING4 | TAOK3 |
| ING4 | FZD5 |
| ING4 | GEMIN5 |
| ING4 | MPHOSPH6 |
| ING4 | CDKN2B |
| ING4 | CNOT4 |
| ING4 | CLCF1 |
| ING4 | NF1 |
| ING4 | ERCC8 |
| ING4 | NLK |
| ING4 | NGF |
| ING4 | CDC27 |
| ING4 | LATS2 |
| ING4 | GADD45A |
| ING4 | RB1 |
| ING4 | JUND |
| ING4 | SMURF2 |
| ING4 | ANAPC4 |
| ING4 | FOXO1 |
| ING4 | IRAK4 |
| ING4 | BIRC6 |
| ING4 | ATR |
| ING4 | APAF1 |
| ING4 | MAFA |
| ING4 | HEATR1 |
| ING4 | CXCL2 |
| ING4 | EIF5B |
| ING4 | NUP153 |
| ING4 | FST |
| ING4 | SMAD5 |
| ING4 | AKT3 |
| ING4 | PAK6 |
| ING4 | UPF2 |
| ING4 | NUP43 |
| ING4 | SPRY2 |
| ING4 | ORC5 |
| ING4 | RPS6KA3 |
| ING4 | SKP2 |
| ING4 | THOC1 |
| ING4 | CASP3 |
| ING4 | CDK1 |
| ING4 | JUN |
| ING4 | RASA1 |
| ING4 | CDK7 |
| ING4 | CBLB |
| ING4 | UBE3A |
| ING4 | NAT10 |
| ING4 | ACACA |
| ING4 | ORC2 |
| ING4 | NVL |
| ING4 | SPRY4 |
| ING4 | SPATA5 |
| ING4 | RPS6KB1 |
| ING4 | ID2 |
| ING4 | MAPK14 |
| ING4 | DCP2 |
| ING4 | RARA |
| ING4 | NUP107 |
| ING4 | PLAUR |
| ING4 | THOC2 |
| ING4 | DCP1B |
| ING4 | NFKB1 |
| ING4 | VEGFA |
| ING4 | E2F4 |
| ING4 | POP1 |
| ING4 | CUL1 |
| ING4 | RIOK1 |
| ING4 | TGS1 |
| ING4 | PAK2 |
| ING4 | FZD8 |
| ING4 | CDKN1B |
| ING4 | SMAD7 |
| ING4 | NUP133 |
| ING4 | ETV4 |
| ING4 | EXOSC3 |
| ING4 | NFAT5 |
| ING4 | FADD |
| ING4 | ITCH |
| ING4 | NUPL1 |
| ING4 | ATM |
| ING4 | RFWD2 |
| ING4 | BRAF |
| ING4 | TAOK1 |
| ING4 | BUB1 |
| ING4 | PER1 |
| HSF1 | ITCH |
| HNF-6 | LSM6 |
| HNF-6 | SOS1 |
| HNF-6 | FANCL |
| HNF-6 | RUNX1 |
| HNF-6 | NFAT5 |
| HNF-3beta | TOB2 |
| HNF-3beta | NCBP1 |
| HNF-3beta | NR1D1 |
| HNF-3beta | BHLHE41 |
| HNF-3beta | TAOK1 |
| HNF-3beta | CNOT6 |
| HES-1 | ANAPC4 |
| GKLF | TOB2 |
| GKLF | ATF2 |
| GKLF | DHX36 |
| GKLF | PRKCI |
| GKLF | TEC |
| GKLF | GEMIN5 |
| GKLF | MPHOSPH6 |
| GKLF | PIM1 |
| GKLF | CNOT4 |
| GKLF | CLCF1 |
| GKLF | NF1 |
| GKLF | ERCC8 |
| GKLF | NLK |
| GKLF | NGF |
| GKLF | ROCK2 |
| GKLF | CDC27 |
| GKLF | LATS2 |
| GKLF | GADD45A |
| GKLF | DBF4 |
| GKLF | LSM6 |
| GKLF | SOS1 |
| GKLF | RB1 |
| GKLF | JUND |
| GKLF | SMURF2 |
| GKLF | SOS2 |
| GKLF | ANAPC4 |
| GKLF | FOXO1 |
| GKLF | IRAK4 |
| GKLF | BIRC6 |
| GKLF | ID4 |
| GKLF | MMP2 |
| GKLF | BIRC3 |
| GKLF | HSPA1A |
| GKLF | APAF1 |
| GKLF | HEATR1 |
| GKLF | KLF3 |
| GKLF | NUP153 |
| GKLF | FST |
| GKLF | SMAD5 |
| GKLF | BMP4 |
| GKLF | UPF2 |
| GKLF | CNOT6L |
| GKLF | SPRY2 |
| GKLF | PHKB |
| GKLF | TRIP12 |
| GKLF | DUSP1 |
| GKLF | RPS6KA3 |
| GKLF | CASP3 |
| GKLF | SOCS3 |
| GKLF | ID3 |
| GKLF | CRK |
| GKLF | DUSP2 |
| GKLF | TSC1 |
| GKLF | TGFB2 |
| GKLF | NR1D1 |
| GKLF | CTGF |
| GKLF | RPS6KA5 |
| GKLF | UBE3A |
| GKLF | CUL4B |
| GKLF | MAP3K1 |
| GKLF | ORC2 |
| GKLF | NVL |
| GKLF | ID1 |
| GKLF | INHBB |
| GKLF | SPRY4 |
| GKLF | NUP160 |
| GKLF | RPS6KB1 |
| GKLF | ID2 |
| GKLF | BMPR1A |
| GKLF | RARA |
| GKLF | NUP107 |
| GKLF | NUP50 |
| GKLF | FRS2 |
| GKLF | PLAUR |
| GKLF | THOC2 |
| GKLF | FOS |
| GKLF | DCP1B |
| GKLF | E2F4 |
| GKLF | CUL1 |
| GKLF | NFKBIE |
| GKLF | RIOK1 |
| GKLF | TGS1 |
| GKLF | PAK2 |
| GKLF | FZD8 |
| GKLF | CDKN1B |
| GKLF | PIK3R3 |
| GKLF | ETV4 |
| GKLF | SMAD2 |
| GKLF | DDX20 |
| GKLF | ITCH |
| GKLF | MAP3K7 |
| GKLF | NUPL1 |
| GKLF | PRKAA2 |
| GKLF | ATM |
| GKLF | BRAF |
| GKLF | TAOK1 |
| GKLF | BUB1 |
| GKLF | PER1 |
| GKLF | CNOT6 |
| Gfi1 | RBL2 |
| GATA | PRKCI |
| GATA | BTRC |
| GATA | CDC27 |
| GATA | SOS1 |
| GATA | NCBP1 |
| GATA | PPP1R3C |
| GATA | CXCL2 |
| GATA | PAK6 |
| GATA | ORC5 |
| GATA | CHUK |
| GATA | RBL2 |
| GATA | RPS6KB1 |
| GATA | ID2 |
| GATA | BMPR1A |
| GATA | PLAUR |
| GATA | CUL1 |
| GATA | RIOK1 |
| GATA | ITCH |
| FPM315(ZNF263) | CLCF1 |
| FPM315(ZNF263) | HSPG2 |
| FPM315(ZNF263) | VEGFA |
| FPM315(ZNF263) | BHLHE41 |
| FPM315(ZNF263) | BHLHE40 |
| Ets | DUSP5 |
| Ets | PRKCI |
| Ets | MMP2 |
| Ets | BIRC3 |
| Ets | UPF2 |
| Ets | THOC1 |
| Ets | RBL2 |
| Ets | TGFB2 |
| Ets | RASA1 |
| Ets | ORC2 |
| Ets | NVL |
| Ets | RARA |
| ER-alpha | ATF2 |
| ER-alpha | NLK |
| ER-alpha | LATS2 |
| ER-alpha | DBF4 |
| ER-alpha | EXOSC2 |
| ER-alpha | IRAK4 |
| ER-alpha | FST |
| ER-alpha | NUP43 |
| ER-alpha | DUSP1 |
| ER-alpha | CASP3 |
| ER-alpha | ID3 |
| ER-alpha | DUSP2 |
| ER-alpha | TGFB2 |
| ER-alpha | SPATA5 |
| ER-alpha | BMPR1A |
| ER-alpha | NUP107 |
| ER-alpha | VEGFA |
| ER-alpha | TGS1 |
| ER-alpha | FZD8 |
| ER-alpha | MAP3K7 |
| ER-alpha | PER1 |
| Egr-1 | PAPD7 |
| Egr-1 | PIM1 |
| Egr-1 | NLK |
| Egr-1 | GADD45A |
| Egr-1 | JUND |
| Egr-1 | SMURF2 |
| Egr-1 | MAFA |
| Egr-1 | PHKB |
| Egr-1 | CASP3 |
| Egr-1 | DUSP2 |
| Egr-1 | RASA1 |
| Egr-1 | CBLB |
| Egr-1 | MAP3K1 |
| Egr-1 | INHBB |
| Egr-1 | RARA |
| Egr-1 | IRS2 |
| Egr-1 | NFKB1 |
| Egr-1 | NUP133 |
| Ebox | HSPG2 |
| E2A | FADD |
| DRI1 | DHX36 |
| DRI1 | TAOK3 |
| DRI1 | PRKCI |
| DRI1 | PIAS1 |
| DRI1 | GEMIN5 |
| DRI1 | CDKN2B |
| DRI1 | CNOT4 |
| DRI1 | CLCF1 |
| DRI1 | NF1 |
| DRI1 | NLK |
| DRI1 | CDC27 |
| DRI1 | LATS2 |
| DRI1 | DBF4 |
| DRI1 | LSM6 |
| DRI1 | RB1 |
| DRI1 | ANAPC4 |
| DRI1 | IRAK4 |
| DRI1 | BIRC6 |
| DRI1 | ATR |
| DRI1 | UBA6 |
| DRI1 | HSPA1A |
| DRI1 | HEATR1 |
| DRI1 | PPP1R3C |
| DRI1 | EIF5B |
| DRI1 | SMAD5 |
| DRI1 | AKT3 |
| DRI1 | PAK6 |
| DRI1 | PIK3CB |
| DRI1 | PER2 |
| DRI1 | SPRY2 |
| DRI1 | FANCL |
| DRI1 | RPS6KA3 |
| DRI1 | SKP2 |
| DRI1 | CHUK |
| DRI1 | RBL2 |
| DRI1 | ID3 |
| DRI1 | CDK1 |
| DRI1 | CDK7 |
| DRI1 | UBE3A |
| DRI1 | CUL4B |
| DRI1 | NAT10 |
| DRI1 | SMAD6 |
| DRI1 | NVL |
| DRI1 | ID1 |
| DRI1 | SPATA5 |
| DRI1 | RPS6KB1 |
| DRI1 | ID2 |
| DRI1 | MAPK14 |
| DRI1 | FRS2 |
| DRI1 | DCP1B |
| DRI1 | IRS2 |
| DRI1 | VEGFA |
| DRI1 | CUL1 |
| DRI1 | NFKBIE |
| DRI1 | RIOK1 |
| DRI1 | PAK2 |
| DRI1 | CDKN1B |
| DRI1 | SMAD7 |
| DRI1 | PIK3R3 |
| DRI1 | NUP133 |
| DRI1 | NFAT5 |
| DRI1 | MAP3K7 |
| DRI1 | ATM |
| DRI1 | BUB1 |
| CRX | DUSP5 |
| CRX | TAOK3 |
| CRX | PRKCI |
| CRX | FZD5 |
| CRX | GEMIN5 |
| CRX | CDKN2B |
| CRX | CLCF1 |
| CRX | NF1 |
| CRX | NLK |
| CRX | CDC27 |
| CRX | LATS2 |
| CRX | DBF4 |
| CRX | LSM6 |
| CRX | EXOSC2 |
| CRX | RB1 |
| CRX | BIRC6 |
| CRX | ID4 |
| CRX | BIRC3 |
| CRX | ATR |
| CRX | NCBP1 |
| CRX | HEATR1 |
| CRX | PPP1R3C |
| CRX | FST |
| CRX | AKT3 |
| CRX | PAK6 |
| CRX | PER2 |
| CRX | UPF2 |
| CRX | NUP43 |
| CRX | CNOT6L |
| CRX | SPRY2 |
| CRX | FANCL |
| CRX | RPS6KA3 |
| CRX | RUNX1 |
| CRX | CASP3 |
| CRX | CHUK |
| CRX | RBL2 |
| CRX | RPS6KA5 |
| CRX | CBLB |
| CRX | NAT10 |
| CRX | ACACA |
| CRX | ORC2 |
| CRX | NVL |
| CRX | NUP160 |
| CRX | RPS6KB1 |
| CRX | DCP2 |
| CRX | FRS2 |
| CRX | FOS |
| CRX | XRN1 |
| CRX | DCP1B |
| CRX | IRS2 |
| CRX | NFKB1 |
| CRX | E2F4 |
| CRX | CUL1 |
| CRX | BHLHE41 |
| CRX | TGS1 |
| CRX | PIK3CA |
| CRX | NFAT5 |
| CRX | FADD |
| CRX | MAP3K7 |
| CRX | NUPL1 |
| CRX | BRAF |
| CRX | BUB1 |
| CREB1 | DHX36 |
| CREB1 | NF1 |
| CREB1 | NCBP1 |
| CREB1 | DUSP1 |
| CREB1 | ID1 |
| CREB1 | BRAF |
| CPBP | RANBP2 |
| CPBP | TOB2 |
| CPBP | ATF2 |
| CPBP | DUSP5 |
| CPBP | DHX36 |
| CPBP | TAOK3 |
| CPBP | FZD5 |
| CPBP | PIAS1 |
| CPBP | PAPD7 |
| CPBP | TEC |
| CPBP | BTRC |
| CPBP | GEMIN5 |
| CPBP | MPHOSPH6 |
| CPBP | PIM1 |
| CPBP | CDKN2B |
| CPBP | CNOT4 |
| CPBP | CLCF1 |
| CPBP | NF1 |
| CPBP | ERCC8 |
| CPBP | NLK |
| CPBP | NGF |
| CPBP | ROCK2 |
| CPBP | CDC27 |
| CPBP | LATS2 |
| CPBP | GADD45A |
| CPBP | DBF4 |
| CPBP | LSM6 |
| CPBP | EXOSC2 |
| CPBP | CNOT1 |
| CPBP | SOS1 |
| CPBP | RB1 |
| CPBP | JUND |
| CPBP | SMURF2 |
| CPBP | SOS2 |
| CPBP | ANAPC4 |
| CPBP | FOXO1 |
| CPBP | ACVR2A |
| CPBP | IRAK4 |
| CPBP | BIRC6 |
| CPBP | ID4 |
| CPBP | MMP2 |
| CPBP | BIRC3 |
| CPBP | ATR |
| CPBP | UBA6 |
| CPBP | HSPA1A |
| CPBP | APAF1 |
| CPBP | MAFA |
| CPBP | NCBP1 |
| CPBP | HEATR1 |
| CPBP | PPP1R3C |
| CPBP | CXCL2 |
| CPBP | KLF3 |
| CPBP | EIF5B |
| CPBP | NUP153 |
| CPBP | FST |
| CPBP | SMAD5 |
| CPBP | PIK3CB |
| CPBP | BMP4 |
| CPBP | PER2 |
| CPBP | UPF2 |
| CPBP | CNOT6L |
| CPBP | SPRY2 |
| CPBP | ORC5 |
| CPBP | PHKB |
| CPBP | TRIP12 |
| CPBP | FANCL |
| CPBP | DUSP1 |
| CPBP | RPS6KA3 |
| CPBP | SKP2 |
| CPBP | THOC1 |
| CPBP | RUNX1 |
| CPBP | CASP3 |
| CPBP | RBL2 |
| CPBP | SOCS3 |
| CPBP | ID3 |
| CPBP | CRK |
| CPBP | DUSP2 |
| CPBP | CDK1 |
| CPBP | TSC1 |
| CPBP | TGFB2 |
| CPBP | JUN |
| CPBP | NR1D1 |
| CPBP | CTGF |
| CPBP | RPS6KA5 |
| CPBP | RASA1 |
| CPBP | CDK7 |
| CPBP | CBLB |
| CPBP | UBE3A |
| CPBP | CUL4B |
| CPBP | MAP3K1 |
| CPBP | NAT10 |
| CPBP | ACACA |
| CPBP | ORC2 |
| CPBP | SMAD6 |
| CPBP | NVL |
| CPBP | ID1 |
| CPBP | INHBB |
| CPBP | SPRY4 |
| CPBP | SPATA5 |
| CPBP | NUP160 |
| CPBP | RPS6KB1 |
| CPBP | ID2 |
| CPBP | UBE3C |
| CPBP | MAPK14 |
| CPBP | DCP2 |
| CPBP | BMPR1A |
| CPBP | RARA |
| CPBP | NUP107 |
| CPBP | NUP50 |
| CPBP | FRS2 |
| CPBP | PLAUR |
| CPBP | THOC2 |
| CPBP | FOS |
| CPBP | HSPG2 |
| CPBP | XRN1 |
| CPBP | DCP1B |
| CPBP | IRS2 |
| CPBP | NFKB1 |
| CPBP | VEGFA |
| CPBP | E2F4 |
| CPBP | POP1 |
| CPBP | CUL1 |
| CPBP | BHLHE41 |
| CPBP | BHLHE40 |
| CPBP | NFKBIE |
| CPBP | RIOK1 |
| CPBP | TGS1 |
| CPBP | PAK2 |
| CPBP | FZD8 |
| CPBP | CDKN1B |
| CPBP | PIK3CA |
| CPBP | SMAD7 |
| CPBP | PIK3R3 |
| CPBP | NUP133 |
| CPBP | CRY2 |
| CPBP | ETV4 |
| CPBP | EXOSC3 |
| CPBP | SMAD2 |
| CPBP | NFAT5 |
| CPBP | DDX20 |
| CPBP | FADD |
| CPBP | ITCH |
| CPBP | MAP3K7 |
| CPBP | NUPL1 |
| CPBP | PRKAA2 |
| CPBP | ATM |
| CPBP | RFWD2 |
| CPBP | BRAF |
| CPBP | TAOK1 |
| CPBP | BUB1 |
| CPBP | PER1 |
| CPBP | CNOT6 |
| CP2 | LSM6 |
| CP2 | BMP4 |
| CP2 | NR1D1 |
| Churchill | RANBP2 |
| Churchill | TOB2 |
| Churchill | ATF2 |
| Churchill | DUSP5 |
| Churchill | DHX36 |
| Churchill | FZD5 |
| Churchill | PIAS1 |
| Churchill | PAPD7 |
| Churchill | TEC |
| Churchill | BTRC |
| Churchill | GEMIN5 |
| Churchill | MPHOSPH6 |
| Churchill | PIM1 |
| Churchill | CNOT4 |
| Churchill | CLCF1 |
| Churchill | NF1 |
| Churchill | ERCC8 |
| Churchill | NLK |
| Churchill | NGF |
| Churchill | ROCK2 |
| Churchill | CDC27 |
| Churchill | LATS2 |
| Churchill | GADD45A |
| Churchill | DBF4 |
| Churchill | EXOSC2 |
| Churchill | SOS1 |
| Churchill | RB1 |
| Churchill | JUND |
| Churchill | SMURF2 |
| Churchill | SOS2 |
| Churchill | ANAPC4 |
| Churchill | FOXO1 |
| Churchill | ACVR2A |
| Churchill | IRAK4 |
| Churchill | BIRC6 |
| Churchill | ID4 |
| Churchill | MMP2 |
| Churchill | ATR |
| Churchill | HSPA1A |
| Churchill | APAF1 |
| Churchill | MAFA |
| Churchill | NCBP1 |
| Churchill | PPP1R3C |
| Churchill | CXCL2 |
| Churchill | KLF3 |
| Churchill | NUP153 |
| Churchill | FST |
| Churchill | SMAD5 |
| Churchill | PIK3CB |
| Churchill | BMP4 |
| Churchill | PER2 |
| Churchill | UPF2 |
| Churchill | CNOT6L |
| Churchill | SPRY2 |
| Churchill | PHKB |
| Churchill | TRIP12 |
| Churchill | DUSP1 |
| Churchill | RPS6KA3 |
| Churchill | SKP2 |
| Churchill | THOC1 |
| Churchill | CASP3 |
| Churchill | SOCS3 |
| Churchill | ID3 |
| Churchill | CRK |
| Churchill | DUSP2 |
| Churchill | TSC1 |
| Churchill | JUN |
| Churchill | RASA1 |
| Churchill | CBLB |
| Churchill | UBE3A |
| Churchill | CUL4B |
| Churchill | MAP3K1 |
| Churchill | NAT10 |
| Churchill | ACACA |
| Churchill | ORC2 |
| Churchill | SMAD6 |
| Churchill | NVL |
| Churchill | ID1 |
| Churchill | INHBB |
| Churchill | SPRY4 |
| Churchill | SPATA5 |
| Churchill | NUP160 |
| Churchill | RPS6KB1 |
| Churchill | ID2 |
| Churchill | UBE3C |
| Churchill | MAPK14 |
| Churchill | DCP2 |
| Churchill | BMPR1A |
| Churchill | RARA |
| Churchill | NUP50 |
| Churchill | FRS2 |
| Churchill | FOS |
| Churchill | XRN1 |
| Churchill | IRS2 |
| Churchill | NFKB1 |
| Churchill | VEGFA |
| Churchill | E2F4 |
| Churchill | POP1 |
| Churchill | CUL1 |
| Churchill | BHLHE41 |
| Churchill | BHLHE40 |
| Churchill | NFKBIE |
| Churchill | RIOK1 |
| Churchill | TGS1 |
| Churchill | PAK2 |
| Churchill | FZD8 |
| Churchill | CDKN1B |
| Churchill | PIK3CA |
| Churchill | SMAD7 |
| Churchill | PIK3R3 |
| Churchill | NUP133 |
| Churchill | CRY2 |
| Churchill | ETV4 |
| Churchill | SMAD2 |
| Churchill | NFAT5 |
| Churchill | FADD |
| Churchill | ITCH |
| Churchill | MAP3K7 |
| Churchill | PRKAA2 |
| Churchill | RFWD2 |
| Churchill | BRAF |
| Churchill | TAOK1 |
| Churchill | PER1 |
| Churchill | CNOT6 |
| CDX-2 | ATF2 |
| CDX-2 | BTRC |
| CDX-2 | CDC27 |
| CDX-2 | DBF4 |
| CDX-2 | LSM6 |
| CDX-2 | CNOT1 |
| CDX-2 | BIRC6 |
| CDX-2 | ID4 |
| CDX-2 | BIRC3 |
| CDX-2 | UBA6 |
| CDX-2 | HSPA1A |
| CDX-2 | MAFA |
| CDX-2 | HEATR1 |
| CDX-2 | PPP1R3C |
| CDX-2 | EIF5B |
| CDX-2 | NUP153 |
| CDX-2 | AKT3 |
| CDX-2 | PAK6 |
| CDX-2 | PER2 |
| CDX-2 | ORC5 |
| CDX-2 | FANCL |
| CDX-2 | DUSP1 |
| CDX-2 | RUNX1 |
| CDX-2 | CASP3 |
| CDX-2 | CHUK |
| CDX-2 | CDK1 |
| CDX-2 | TGFB2 |
| CDX-2 | JUN |
| CDX-2 | CTGF |
| CDX-2 | RPS6KA5 |
| CDX-2 | CUL4B |
| CDX-2 | ID1 |
| CDX-2 | UBE3C |
| CDX-2 | BMPR1A |
| CDX-2 | NUP107 |
| CDX-2 | FRS2 |
| CDX-2 | PLAUR |
| CDX-2 | THOC2 |
| CDX-2 | FOS |
| CDX-2 | XRN1 |
| CDX-2 | NFKB1 |
| CDX-2 | E2F4 |
| CDX-2 | POP1 |
| CDX-2 | CUL1 |
| CDX-2 | BHLHE41 |
| CDX-2 | RIOK1 |
| CDX-2 | FZD8 |
| CDX-2 | CRY2 |
| CDX-2 | ITCH |
| CDX-2 | MAP3K7 |
| CDX-2 | PRKAA2 |
| BRCA1:USF2 | CNOT4 |
| BRCA1:USF2 | BIRC6 |
| BRCA1:USF2 | TGFB2 |
| BRCA1:USF2 | NUP107 |
| BRCA1:USF2 | PLAUR |
| BEN | TOB2 |
| BEN | CDC27 |
| BEN | MMP2 |
| BEN | PPP1R3C |
| BEN | RPS6KA3 |
| BEN | BMPR1A |
| BEN | NFKB1 |
| AP-1 | PIM1 |
| AP-1 | NLK |
| AP-1 | PLAUR |
| AML3 | EIF5B |
| AML3 | RUNX1 |
| AML3 | CHUK |
| AML3 | ATM |
| AML3 | PER1 |
| AHR | RANBP2 |
| AHR | TOB2 |
| AHR | MPHOSPH6 |
| AHR | CDKN2B |
| AHR | ERCC8 |
| AHR | CDC27 |
| AHR | GADD45A |
| AHR | SOS1 |
| AHR | SMURF2 |
| AHR | ANAPC4 |
| AHR | BIRC6 |
| AHR | MMP2 |
| AHR | BIRC3 |
| AHR | NCBP1 |
| AHR | KLF3 |
| AHR | PER2 |
| AHR | CNOT6L |
| AHR | SPRY2 |
| AHR | RPS6KA3 |
| AHR | SKP2 |
| AHR | CASP3 |
| AHR | RBL2 |
| AHR | SOCS3 |
| AHR | CRK |
| AHR | CDK1 |
| AHR | TSC1 |
| AHR | TGFB2 |
| AHR | JUN |
| AHR | NR1D1 |
| AHR | RPS6KA5 |
| AHR | RASA1 |
| AHR | MAP3K1 |
| AHR | NAT10 |
| AHR | ACACA |
| AHR | ORC2 |
| AHR | INHBB |
| AHR | SPRY4 |
| AHR | ID2 |
| AHR | UBE3C |
| AHR | DCP2 |
| AHR | NUP107 |
| AHR | PLAUR |
| AHR | FOS |
| AHR | NFKB1 |
| AHR | POP1 |
| AHR | CUL1 |
| AHR | BHLHE41 |
| AHR | NFKBIE |
| AHR | PAK2 |
| AHR | FZD8 |
| AHR | ETV4 |
| AHR | EXOSC3 |
| AHR | SMAD2 |
| AHR | MAP3K7 |
| AHR | NUPL1 |
| AHR | PRKAA2 |
| AHR | ATM |
| AHR | RFWD2 |
| AHR | BRAF |
| AHR | CNOT6 |
