# Supplementary material for: Data on the gene expression of cardiomyocyte exposed to hypothermia
Source: Data Brief. 2016 May 11;8:45–8. doi: 10.1016/j.dib.2016.04.061 (PMC4885011; doi:10.1016/j.dib.2016.04.061)
Supplement: Supplementary file 2 — Supplementary material [file mmc2.zip › supplementary data/Supplementary table 4.doc]

Table.4 Sequence of PCR Primers Used in This Study Gene Forward primer Reverse primer:

| Gene | Forward primer | Reverse primer |
| --- | --- | --- |
| DKK1 | CCTTGAACTCGGTTCTCAATTCC | CAATGGTCTGGTACTTATTCCCG |
| SMAD7 | TTCCTCCGCTGAAACAGGG | CCTCCCAGTATGCCACCAC |
| RHOB | GGGGCTTATCCGCACTTACC | TGGCCTGATACTCGGTGAACA |
| KLF5 | CCTGGTCCAGACAAGATGTGA | GAACTGGTCTACGACTGAGGC |
| SOCS4 | GCCGACAGAAAAGACGGTTAT | GGTTCCTTAAAGACACTTCGGTT |
| CDK1 | AAACTACAGGTCAAGTGGTAGCC | TCCTGCATAAGCACATCCTGA |
| SUDS3 | GAGTCGGACGAAGACACTGAG | TGCATTCCGTATCCTCTCTTTGT |
| ZNF17 | TTTAGATGTCGCTCCACACTTG | TGCCGAATGAGGTGGGAATTT |
| MED23 | GGGCCTTCAGACAGTTTTGG | AGCTGTGTTCTTTCCCACTCA |
| ZDHHC17 | GATGTACGGCAACCGGACAAA | TGATCCACAATAGCACCTTTCG |
| SOS2 | ATGTAGAGGAGCGAGTTCAGAA | ATGGTAGTCCACTTTGTACCCT |
| 18S | CGGCTACCACATCCAAGGAA | GCTGGAATTACCGCGGCT |
